# Very different performance of the power Doppler modalities of several ultrasound machines ascertained by a microvessel flow phantom

**DOI:** 10.1186/ar4345

**Published:** 2013-10-24

**Authors:** David F Ten Cate, Jolanda J Luime, Myrthe van der Ven, Johanna MW Hazes, Klazina Kooiman, Nico de Jong, Johannes G Bosch

**Affiliations:** 1 Department of Rheumatology, Erasmus Medical Center Rotterdam, Dr. Molewaterplein 50-60, Rotterdam 3015 GE, The Netherlands; 2 Department of Biomedical Engineering, Erasmus Medical Center Rotterdam, Dr. Molewaterplein 50-60, Rotterdam 3015 GE, The Netherlands

## Abstract

**Introduction:**

In many patients with rheumatoid arthritis (RA) subclinical disease activity can be detected with ultrasound (US), especially using power Doppler US (PDUS). However, PDUS may be highly dependent on the type of machine. This could create problems both in clinical trials and in daily clinical practice. To clarify how the PDUS signal differs between machines we created a microvessel flow phantom.

**Methods:**

The flow phantom contained three microvessels (150, 1000, 2000 microns). A syringe pump was used to generate flows. Five US machines were used. Settings were optimised to assess the lowest detectable flow for each US machine.

**Results:**

The minimal detectable flow velocities showed very large differences between the machines. Only two of the machines may be able to detect the very low flows in the capillaries of inflamed joints. There was no clear relation with price. One of the lower-end machines actually performed best in all three vessel sizes.

**Conclusions:**

We created a flow phantom to test the sensitivity of US machines to very low flows in small vessels. The sensitivity of the power Doppler modalities of 5 different machines was very different. The differences found between the machines are probably caused by fundamental differences in processing of the PD signal or internal settings inaccessible to users. Machines considered for PDUS assessment of RA patients should be tested using a flow phantom similar to ours. Within studies, only a single machine type should be used.

## Introduction

Rheumatoid arthritis (RA) is a common disease with a prevalence of around 1% worldwide [1]. RA is potentially an invalidating disease [2], but early diagnosis in the so-called window of opportunity [3,4] and treatment according to a treat-to-target protocol [5] can optimise the outcome for RA patients. Adding ultrasound (US) to the diagnostic workup and monitoring of treatment may provide even better results. In rheumatological US both greyscale and power Doppler (PD) are used, of which PDUS seems to have the largest value. PDUS has the potential to reclassify patients to a higher joint group according to the 2010 classification criteria for RA, increasing the risk for undifferentiated arthritis to be definite RA [6]. Furthermore, the presence of PDUS inflammation in joints that are not swollen at clinical examination has shown to be clinically relevant in patients in remission of RA, since it predicts occurrence of flare and erosive progression [7-10]. Correct assessment of the presence and absence of a PD signal indicating the presence of inflammation is therefore vital in rheumatological US.

**Figure 1:**
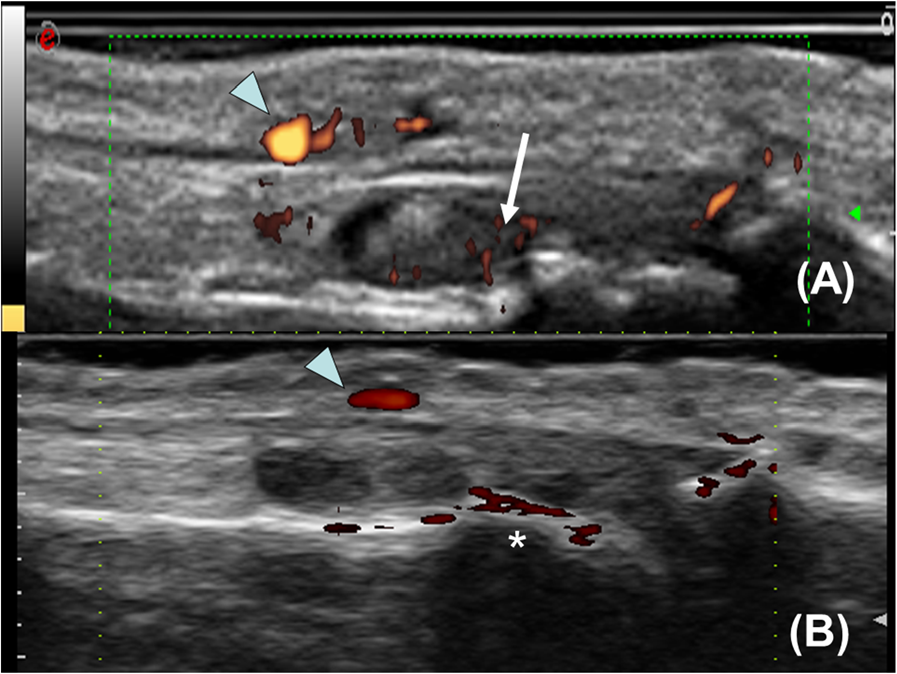
**Different performance of the power Doppler ultrasound modality of two machines in one patient. (A)** Machine B, presence of a positive power Doppler signal (arrow) within the region of synovial proliferation.** (B)** Machine A, this signal is absent. Arrowhead, vessel; *noise on cortical surface.

However, PDUS may be highly dependent on the type of US machine used [11,12]. We also observed this in our centre (see Figure 1).

If there are indeed large differences in the performance of PDUS per machine, the choice of machine might be essential for a valid detection of inflammation. Using different machines within a multi-machine study or during patient treatment could then have a detrimental impact on treatment decisions or study outcome.

To quantify the suspected differences in PD sensitivity of different machines in an objective way, we decided to perform an *in vitro* experiment. To compare the PD function of different US machines one could use a flow phantom. This flow phantom should mimic the tissue that is scanned by PDUS in rheumatology; that is, very small vessels and very low flows. To our knowledge, no studies have been conducted investigating the size of capillaries and the blood flow velocity in an inflamed joint, but there are data on capillaries in healthy subjects’ nailfolds and capillaries in periulcerous regions. These capillaries have a diameter of around 30 μm and the blood flow velocity can be as low as 0.5 mm/second [13,14]. Flow phantoms previously presented did not compare US machines [15], used vessels that were considerably larger than capillaries, or assessed many capillaries close to each other at once, making it impossible to evaluate the flow velocity in the individual vessels [11,12,16,17]. For these reasons, we created a new flow phantom with a very small, single vessel to obtain the lowest detectable flow velocity of five US machines. Two additional larger vessels were included in the phantom for comparison with the literature [11,12].

## Methods

### Phantom

**Figure 2:**
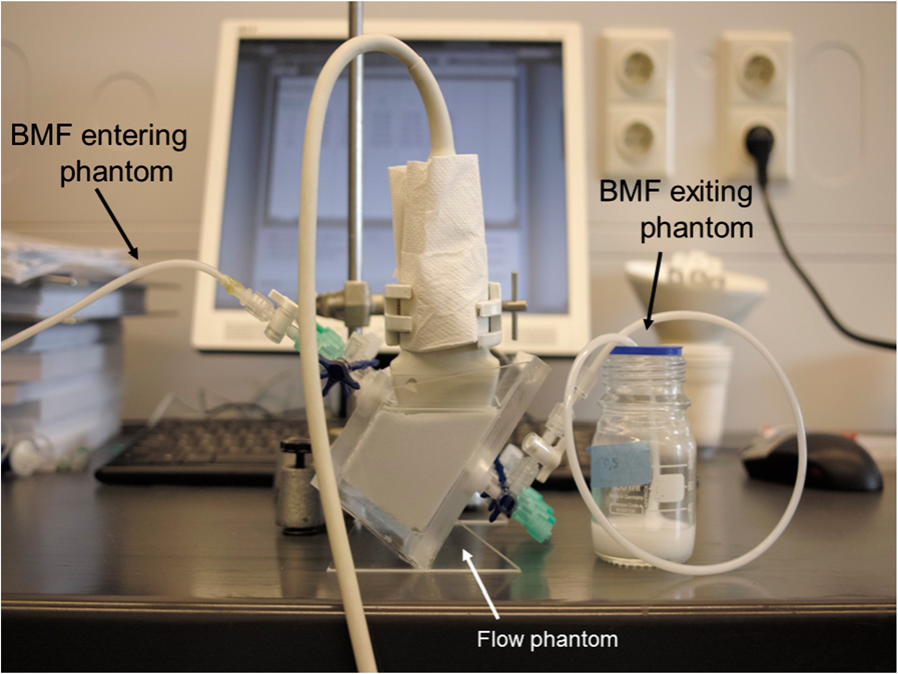
**Flow phantom with a fixated probe.** BMF, blood-mimicking fluid.

The flow phantom (Figure 2) consisted of an acrylic (polymethyl methacrylate) container filled with tissue-mimicking material, according to a previously published recipe [18]. In this tissue-mimicking material we placed three microvessels: 150 μm (inner diameter) vessel made of polyethylene terephthalate glycol-modified (Paradigm Optics, Vancouver, WA, USA), and 2,000 μm and 1,000 μm (inner diameter) vessels made of silicone (Eriks bv, Alkmaar, the Netherlands). These two vessels were included to compare our phantom with already published studies [11,12]. Initially we used vessels with diameters of 50 μm and 100 μm also made of polyethylene terephthalate glycol-modified, but these were blocked almost instantly. The blood-mimicking fluid (BMF) was based on the recipe by Ramnarine and colleagues [19]. Briefly, 91.07% (w/w) demineralised water, 1.18% (w/w) dextran (average 150 kDa, D4876; Sigma-Aldrich, Zwijndrecht, the Netherlands), 0.90% (w/w) ICI supersonic N surfactant, 5.03% (w/w) glycerol, and 1.82% w/w orgasol particles (5 μm in diameter; Arkema B.V., Rotterdam, The Netherlands) were mixed using a magnetic stirrer. The BMF was then filtered using a 40 μm sieve (352340; BD, Breda, the Netherlands) and degassed using a vacuum pump. Compared with the original recipe by Ramnarine and colleagues, our BMF contained half the amount of dextran and glycerol – this made our BMF less viscous, which was necessary to prevent blockage of the vessels. A syringe pump (Hugo Sachs Elektronik, March-Hugstetten, Germany) was used to generate flows. This pump can produce regular flows as low as 1.28 pl/minute. For each vessel size, flow settings (ml/hour) were calculated that corresponded to average flow velocities ranging from 40 to 0.005 mm/second, using the following equation, where *Q* is flow (m^3^/second), *V*_avg_ is the average flow velocity (m/second) and *R* is the radius (m):

$$Q=V_{\mathrm{avg}}\times \pi R^{2}$$

The actual volume transported through the vessels was tested by turning on the pump, and completely filling the vessel until drops of BMF came out of the capillary. A complete number of drops were captured in a container while recording the time. This container was weighed before and after this experiment on a microbalance. With the relative density of the BMF we calculated the flow (transported volume per time).

### Experiment

**Figure 3:**
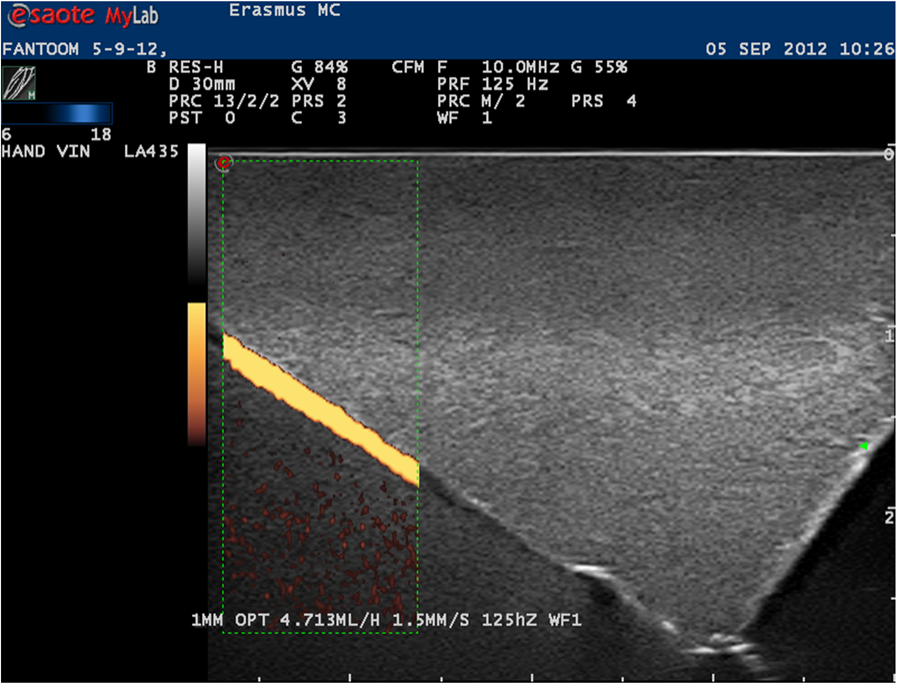
Continuous PDUS signal in a 1 mm vessel on Machine B.

The lowest detectable flow for each machine/vessel diameter combination was defined as the flow that still resulted in a continuous PDUS signal (Figure 3). First the pump was set to a high flow, and then gradually decreased in steps until a continuous PD signal could just still be detected. The value of the lowest flow was recorded. Between each change of pump flow we waited 5 minutes for the system to reach stable flow velocities. For each lowest detectable flow per vessel we stored an image and recorded the machine settings used to acquire this image.

### Ultrasound machines and settings

Five available US machines were tested (Table 1). Machines A and B are used in our Department of Rheumatology in daily clinical practice. Machine C is a high-end machine for general imaging. Machines D and E are specialised research machines, the latter a highly specialised machine for high-frequency small animal imaging. Four machines operated at or around the most common frequency of 10 MHz (Machines A, B, D and E), and one (Machine C) operated lower at a frequency between 3 and 9 MHz (actual frequency not displayed on this machine). Settings on all machines were optimised to detect lowest flows by adjusting pulse repetition frequency (PRF)/velocity range, wall filters, Doppler frequency and Doppler gain. In general this meant for all vessels using the lowest wall filter, the lowest velocity range or PRF and the highest suitable Doppler gain with respect to noise level. One experienced musculoskeletal ultrasonographer (DFTC) performed all US examinations.

**Table 1:** Machines tested (alphabetical order) and probes used

Machine A	Aloka Ltd., Tokyo, Japan α7 (probe UST-5411)
Machine B	Esaote, Maastricht, the Netherlands MyLab60 (probe LA 435)
Machine C	Philips Healthcare, Amsterdam, the Netherlands iU22 (probe L9-3)
Machine D	Ultrasonix Medical Corporation, Vancouver, Canada (probe L14-5/38)
Machine E	VisualSonics, Toronto, Canada Vevo2100 (probe MS200)

## Results

We found that the pump was accurate enough for our purposes, especially when taking into account the very low flows used (see Table 2). The lowest detectable flow velocities in the different vessels are presented in Table 3. These differed very much, by a factor of 100 between machines. This was the case for all vessel sizes. In the smallest vessel (150 μm), which most resembles the situation in an inflamed joint, two machines (Machines D and E) could not detect a positive PD signal at all at any flow velocity. For the other vessels, the minimal detectable velocity ranged from 0.11 mm/second (Machine B) to 11.1 mm/second (Machine A). The settings were optimised for the detection of lowest flow. See Table 4 for the settings of PRF/velocity range, wall filter and Doppler frequency per machine for the smallest vessel.

**Table 2:** Measuring the reliability of the pump

	**2,000 μm vessel**	**1,000 μm vessel**	**150 μm vessel**	**150 μm vessel**	**150 μm vessel**
Set flow	11.31	3.142	0.141	0.070	0.035
Measured flow	10.68	2.948	0.276	0.108	0.049

Flow (ml/hour).

**Table 3:** Lowest detected flow velocity resulting in a continuous positive power Doppler ultrasound signal

**Machine**	**2,000 μm vessel**	**1,000 μm vessel**	**150 μm vessel**
A	4	2.22	11.1
B	0.005	0.06	0.11
C	1	0.56	1.68
D	1	0.56	Not detected
E	0.5	0.33	Not detected

Flow velocity (mm/second).

**Table 4:** Settings for detection of lowest flow velocity in the 150 μm vessel

**Machine**	**Pulse repetition frequency/velocity range**	**Wall filter**	**Doppler frequency**
A	1.3 cm/second	Level 1	8 MHz
B	125 Hz	Level 1	10 MHz
C	150 Hz	15 Hz	R1^a^
D^b^	200 Hz	Level 1	10 MHz
E^b^	1,000 Hz	Low	12.5 MHz

^a^Actual frequency not displayed on this machine. ^b^No flow detected in this vessel.

## Discussion

We showed that the sensitivity of the PD modalities of five US machines (three machines used in the clinic and two used for research) was very different, using a microvessel flow phantom. The very large differences found between the machines are only partly explained by each machine’s Doppler frequency, lower limits of PRF and wall filter settings, but are most probably caused mainly by fundamental differences in processing the PD signal or internal settings inaccessible to users. There was no clear relation with price or technical sophistication of the machines: a lower-end machine (Machine B) performed best for all three vessels, while mid-range and high-end research machines (Machines D and E) did not detect any flow in the smallest vessel, against expectations.

Only one machine of the five (Machine B) could detect the low flow velocity in capillaries that based on previous research are estimated to be between 0.5 and 1 mm/second. Machine C came close to this limit, which underlines our conclusion that the observed differences are mainly caused by differences in processing of the signal, since the probe that was available for Machine C had a bandwidth of only 3 to 9 MHz. When a high-frequency probe would have been used with this machine, it might also have been able to detect flow less than 1 mm/second in the smallest vessel. The other machines did not perform appropriately according to this limit.

As mentioned above, flow phantoms have been published in the literature before [11,12,15-17]. However, when comparing the PD modalities of different US machines it is essential to use small, individual vessels. A positive PD signal depends on the total detected Doppler signal power within the range gate (the colour Doppler pixel size, typically <1 mm). This power depends on the number of particles that have a velocity above a certain threshold. This threshold is determined by the wall filter, the PRF/velocity range. Whether a PD signal is actually detected/displayed is also dependent on the noise level of the system and the system’s ability to suppress clutter and signal from stationary targets. If the vessel diameter is larger than the gate size, the velocity threshold will determine the lowest detectable velocity. This explains why the minimum velocities found for 1 and 2 mm vessels are similar.

However, if the velocity is the same but the vessel is much smaller than the gate size, the number of moving particles will be lower and more stationary tissue will be inside the gate range. The tissue suppression and noise level then become more important and the minimal detectable velocity will be raised. This means that a phantom with a vessel too large in diameter [11,12] may use a flow velocity similar to that in vessels in an inflamed joint, but more particles are inside one pixel in the phantom situation (*in vitro*) compared with the situation in an inflamed joint (*in vivo*). This can possibly cause a positive PD signal based on the large number of particles. In a flow phantom using a bundle of capillaries [16,17] one can never know for sure the flow velocity in each vessel. The possibility therefore remains that the flow is very high in a few capillaries, causing a positive PD signal solely based on the high flow velocity of particles in these few capillaries.

A study comparing Machines A and B (older versions than in our study) on a 1,000 μm flow phantom has been published in the past [12]. These older versions of the machines were ranked regarding sensitivity the same as in our study. However, in our study Machine B detected a considerably lower flow compared with the previous study: 0.06 mm/second in our study versus 1.3 mm/second in the previous. Machine A detected a twofold lower flow in our study: 2.2 mm/second in our study versus 3.9 mm/second in the earlier study.

Another study tested an earlier, single-element version of Machine E (VisualSonics, Toronto, Canada Vevo 770;) on a microvessel flow phantom with vessel dimensions similar to ours (160 μm) [15]. In this microvessel, the Vevo 770 did detect flows as low as 0.5 mm/second. In our study Machine E did not detect any flow in the smallest vessel (150 μm). A possible explanation for this higher sensitivity for low flows could be that the Vevo 770 uses a mechanically steered probe with a single element, as opposed to the array probe we used on Machine E in our study. In general, the Doppler processing of a single-element system can be very different from that of an array system.

Some observations raised discussion within our research group. One of these discussions was about the very low flows detected by Machine B in the 2,000 μm vessel. To verify this finding the experiment was repeated several times by two observers (DFTC and MvdV), which resulted in similar findings. When setting the flow slightly lower, the signal disappeared. Therefore, we think the measured flow is correct. A possible explanation for this low limit is that the PRF can be set to a very low level and the wall filter cutoff frequency is probably also very low, in combination with a good clutter suppression. However, in *in vivo* situations, normal tissue or probe motion will prevent detecting such extremely low flows.

Another observation that raised discussion is the lower flow detected in the 1,000 μm vessel as compared with the 2,000 μm vessel. A reason for this could be that the flow velocity profile in the 1,000 μm vessel is shaped differently, as compared with the 2,000 μm vessel, resulting in a larger difference between average flow and maximum flow. This may even have been reinforced by compression of the smaller vessel by the tissue-mimicking material. This means the average flow velocity is actually higher than estimated, since the calculation is quadratically dependent on the microvessel diameter. If the maximum velocity of the peak flow is slightly higher than the wall filter cutoff, this results in a positive PD signal. The peak flow may therefore be rather similar in the 2,000 μm and the 1,000 μm vessels, but due to the shape of the flow profile this corresponds to a lower average flow velocity in the 1,000 μm vessel. While the true value for the flow velocities may differ from the calculated values, this difference is the same for all machines, so the comparison between machines is still valid per vessel.

A drawback of our study is that we have made assumptions on the capillary sizes and flow velocities in inflamed joints based on papers published on healthy subjects and periulcerous regions. This may not be entirely correct. Therefore, at present it is crucial to ascertain the flow velocities and capillary sizes in inflamed joints. With this information the minimal flows that rheumatological US machines need to be able to detect will be known.

Nonetheless, for a reliable and reproducible detection of very low flows in inflamed joints, the choice of the US machine and its settings seems very important. Caution should be exercised when conducting a multi-machine trial or when making treatment decisions based on PDUS. Our flow phantom could be used to decide which US machine to use both in clinical practice and in clinical trials.

## Conclusions

We created a flow phantom to test the sensitivity of US machines to very low flows in small vessels. We found that the sensitivity of the PD modalities was very different between five US machines. Based on the results of our study it would be advisable to standardise and validate US machines both for rheumatological clinical practice and for clinical trials. Our phantom could be used for this purpose.

## Abbreviations

BMF: Blood-mimicking fluid; PD: Power Doppler; PRF: Pulse repetition frequency; RA: Rheumatoid arthritis; US: Ultrasound.

## Competing interests

The authors declare that they have no competing interests.

## Authors’ contributions

All authors participated in the conception and design of this study and interpretation of the data. DFTC, MvdV and JGB performed the data acquisition. MvdV and KK made the blood mimicking fluid. DFTC, MvdV and JGB performed the data analysis. All authors were involved in drafting the article or revising it critically for important intellectual content. JJL, JMWH and NdJ have been focused primarily on the conception and design of the study and the interpretation of the data. Of course they were also very much involved in drafting the article and revising it critically. All authors read and approved the final manuscript. 
